# Hantavirus Pulmonary Syndrome in Traveler Returning from Nepal to Spain

**DOI:** 10.3201/eid2601.181685

**Published:** 2020-01

**Authors:** Elena Sulleiro, Maria Luisa Aznar, Núria Serre-Delcor, Fernando Salvador, Adrian Sanchez-Montalvá, Mateu Espasa, Daniel Molina, Fernando de Ory, M. Paz Sanchez-Seco, Israel Molina, Candido Diaz-Lagares, Miguel J. Martinez, Tomàs Pumarola, Inés Oliveira

**Affiliations:** Vall d’Hebrón University Hospital, PROSICS Barcelona, Universitat Autònoma de Barcelona, Barcelona, Spain (E. Sulleiro, M.L. Aznar, N. Serre-Delcor, F. Salvador, A. Sanchez-Montalvá, M. Espasa, D. Molina, I. Molina, C. Diaz-Lagares, T. Pumarola, I. Oliveira);; Instituto de Salud Carlos III, Majadahonda, Spain (F. de Ory, M.P. Sanchez-Seco);; Hospital Clínic, Barcelona (M.J. Martinez)

**Keywords:** hantavirus, cardiopulmonary syndrome, Nepal, traveler, India, Sri Lanka, European arvicoline hantaviruses, American sigmodontine hantaviruses, respiratory infections, viruses, zoonoses, Spain

## Abstract

Most human hantavirus infections occur in Asia, but some cases have been described in Europe in travelers returning from Asia. We describe a case of hantavirus pulmonary syndrome in a previously healthy traveler occurring shortly after he returned to Spain from Nepal. Serologic tests suggested a Puumala virus–like infection.

More than 24 pathogenic hantaviruses that are known to be pathogenic to humans have been identified worldwide (*1*). The diseases these viruses have caused have been traditionally divided into 2 major clinical syndromes: hemorrhagic fever with renal syndrome (HFRS) in Europe, Asia, and Africa, and hantavirus pulmonary syndrome (HPS; sometimes referred to as hantavirus cardiopulmonary syndrome) in the Americas (*2*). We describe a life-threatening hantavirus infection in a patient with respiratory failure returning to Spain from Nepal. 

In October 2017, a 28-year-old man sought care at the outpatient clinic of Drassanes Tropical Medicine Unit at Hospital Vall d’Hebrón, Barcelona, Spain, on day 2 after onset of symptoms. He had recently returned from a 5-week trip to Nepal (Appendix), during which he stayed at basic hostels, where sounds of rats or mice were audible. The patient reported onset of fever, malaise, weakness, headache, arthromyalgia, and abdominal pain during his flight back to Spain. Medical examination revealed only fever (38.2°C) and a mild diffuse macular rash on his trunk. Laboratory tests showed thrombocytopenia (platelets 125 × 10^9^/L) and mild liver enzyme elevation (alanine aminotransferase 127 IU/L, aspartate aminotransferase 81 IU/L); results of tests for various pathogens, including hantavirus, were negative (Table).

**Table:** Clinical and laboratory findings over time of a patient with hantavirus pulmonary syndrome after travel, Spain*

Findings	Acute phase, day 2	ICU admission, day 6	ICU discharge, day 12	Follow-up, day 22	Follow-up, day 82
Clinical	Fever, myalgia, headache, vomiting, and diarrhea	Acute noncardiac pulmonary edema and hypotension; pleural effusions and respiratory failure	Eupneic, resolution of the pulmonary edema	Mild dyspnea general condition with fatigue	Completely recovered
Hematalogic	Leukocytes 4.38 × 10^9^ cells/L; platelets 125 × 10^9^/L	Leukocytes 6.17 × 10^9^ cells/L; platelets 92 × 10^9^/L	Leukocytes 7.00 × 10^9^ cells/L; platelets 306 × 10^9^/L	Leukocytes 4.91 × 10^9^ cells/L; platelets 205 × 10^9^/L	Leukocytes 5.82 × 10^9^ cells/L; platelets 208 × 10^9^/L
Biochemical	AST 81 IU/L; ALT 127 IU/L; ALP 142 IU/L; GGT 128 IU/L; creatinine 1.02 g/dL; urea 37 mg/dL; Na^+ ^140 mmol/L; K^+ ^4.24 mmol/L; cholesterol 113 mg/dL; triglycerides 78 mg/dL; CRP 10.33	AST 448 IU/L; ALT 424 IU/L; ALP 151 IU/L; GGT 150 IU/L; cardiac biomarkers (troponin and natriuretic peptide) normal; creatinine 0.86 mg/dL; urea 53 mg/dL; Na^+ ^135.6 mmol/L; K^+ ^4.39 mmol/L; LDH 830 UI/L; CRP 14.58	AST 117 IU/L; ALT 205 IU/L; Creatinine 0.56 mg/dL; Urea 28 mg/dL; Na^+^ 141.8 mmol/L; K^+^ 4.38 mmol/L; cholesterol 121 mg/dL; triglycerides 218 mg/dL; CRP 2.81	AST 46 IU/L; ALT 106 IU/L; ALP 176 IU/L; GGT 143 IU/L; creatinine 0.71 mg/dL; Urea 30 mg/dL; Na^+^ 139.5 mmol/L; K^+^ 4.58 mmol/L	AST 24 IU/L; ALT 19 IU/L; GGT 12 IU/L; creatinine 0.95 mg/dL; urea 33 mg/dL; Na^+^ 140.2 mmol/L; K^+^ 4.78 mmol/L
Arterial blood gas analysis	NA	pH 7.36; PaCO_2_ 32 mm Hg; PaO_2_ 40 mm Hg; SaO_2_ 85.5%; HCO_3-_ 26.3 mm Hg	NA	NA	NA
Pleural effusion characteristics	NA	Glucose 114 mg/dL; proteins 2.1 g/dL; LDH 423 IU/L; cytology inflammatory process with lymphocyte predominance	NA	NA	NA
Hantavirus diagnosis	IgG (ELISA) negative; IgG (IBT) negative; IgM (ELISA) negative; IgM (IBT) negative; PCR (serum + urine) negative	NA	IgG (ELISA) weak positive; IgG (IBT) positive, PUUV indeterminate, Dobrava Seoul, Hantaan, Sin Nombre and Andes negative; IgM (ELISA) indeterminate; IgM (IBT) negative; PCR (serum) negative; DENV IgG, IgM negative, PCR (serum) negative	IgG (ELISA) positive; IgG (IBT) positive; PUUV indeterminate; Dobrava, Sin Nombre and Andes and negative Seoul and Hantaan; IgM (ELISA) positive; IgM (IBT) negative; PCR (serum) negative	IgG (ELISA) indeterminate; IgG (IBT) indeterminate; PUUV, Dobrava, and Seoul negative Hantaan, Sin Nombre and Andes; IgM (ELISA) negative; IgM (IBT) negative; PCR (serum) negative

*Days are days after initial symptom onset. An expanded table including additional results is available (https://wwwnc.cdc.gov/EID/article/26/1/18-1685-T1.htm). ALP, alkaline phosphatase; ALT, alanine transaminase; AST, aspartate transaminase; CRP, C-reactive protein; DENV, dengue virus; GGT, gamma-glutamyl transpeptidase; IBT, immunoblot; ICU, intensive care unit; LDH, lactate dehydrogenase; NA, not applicable; PUUV, Puumala virus.

The patient returned home, but on day 4, he incurred a head wound after a fall caused by dizziness, requiring treatment at the Vall d’Hebrón Hospital emergency department; he was discharged on day 5 in stable condition and with normal chest radiograph results. On day 6, he was readmitted for dyspnea; within 12 hours, respiratory failure developed, requiring admission to the intensive care unit with high-flow nasal cannula oxygen therapy and vasoactive support. Results of a new chest radiograph revealed bilateral pleural effusion and extensive alveolar edema. Electrocardiogram results showed no abnormality, but echocardiogram results showed mild ventricular dysfunction (left ventricular ejection fraction 50%) without evidence of pericardial effusion. 

The patient required respiratory and vasoactive support in the intensive care unit for 5 days and he was then transferred to a regular room. At that point, 12 days after symptom onset, repeat testing showed positive results for hantavirus IgM (weak positive) and IgG (Table). The diagnosis was established as HPS, as defined by the US Centers for Disease Control and Prevention (CDC) classification (*3*). The patient was discharged to his home 2 days later. Repeat test results on day 22 after symptom onset remained positive for hantavirus IgM and IgG. A month after discharge, the patient still reported a mild dyspnea and fatigue. Pulmonary function tests showed no abnormality. Intolerance to exercise lasted for 2 months after discharge. One year later, he is fully recovered without sequelae.

The course of this patient was as classically described for HPS: an initial prodromal phase with influenza-like symptoms, followed by a rapid progression to abrupt onset of respiratory failure (*3*). Elevated levels of C-reactive protein and lactate dehydrogenase were found soon after symptom onset (Table), as described in the literature (*4*). Results of coagulation, cardiac enzyme, and renal function tests were normal throughout hospitalization, but proteinuria and microhematuria were not evaluated. 

Once the hantavirus diagnosis was established, we contacted the patient’s trip partner, who accompanied him during the first 2 weeks in Nepal. He was asymptomatic; results of a serologic test (ELISA) performed 7 weeks after his return was negative for hantavirus IgM and IgG.

Little is known about the incidence of hantavirus infections in Nepal. Thottapalayam virus, a genetically distant virus from other Old World hantaviruses, has been detected in the Asian house shrew (*Suncus murinus*) (*5*); no human cases of infection with this virus have been reported. However, serologic evidence of hantavirus infection in patients with fever of unknown origin has been reported in Nepal (*6*). Serum and urine samples from our patient tested negative by an in-house nested reverse transcription PCR (RT-PCR) targeting the small segment of the viral genome for detection of New World and Old World hantaviruses. Viral RNA is rarely found in blood more than a few days after onset of fever, and similar negative results in RT-PCR have been previously reported (*7*–*9*). Alternatively, the infection may have been caused by a genetically different hantavirus not detected by the in-house hantavirus RT-PCR. Furthermore, the serum sample was frozen and thawed several times, which may have degraded the RNA.

The pattern of serologic findings able to confirm only a transitory presence of Puumala virus (PUUV) IgG suggest a cross-reaction with an unknown hantavirus, because the known PUUV reservoir, the *Myodes glareolus* bank vole, is absent from Nepal and India. The PUUV IgG and IgM seroconversion and the classical HPS manifestation (*3*) are highly reminiscent of 2 fatal HFRS/HPS cases previously described in South India (*8*,*9*) that were also PUUV immunoblot positive.

Hantaviruses are emerging zoonotic pathogens and, although recognition of the infection in humans has greatly improved worldwide during the past decade, many cases probably remain undiagnosed. This case highlights the importance of clinical suspicion of hantavirus infection in travelers, even in countries where no cases have been previously reported.

Appendix Additional information about hantavirus pulmonary syndrome identified in a traveler to Nepal on his return to Spain.
