# Supplementary material for: Hantavirus Pulmonary Syndrome in Traveler Returning from Nepal to Spain
Source: Emerg Infect Dis. 2020 Jan;26(1):150–3. doi: 10.3201/eid2601.181685 (PMC6924883; doi:10.3201/eid2601.181685)
Supplement: Appendix — Additional information about hantavirus pulmonary syndrome identified in a traveler to Nepal on his return to Spain. [file 18-1685-Techapp-s1.pdf]

Article DOI: https://doi.org/10.3201/eid2601.181685

Hantavirus Pulmonary Syndrome in Traveler Returning from Nepal to Spain

Appendix

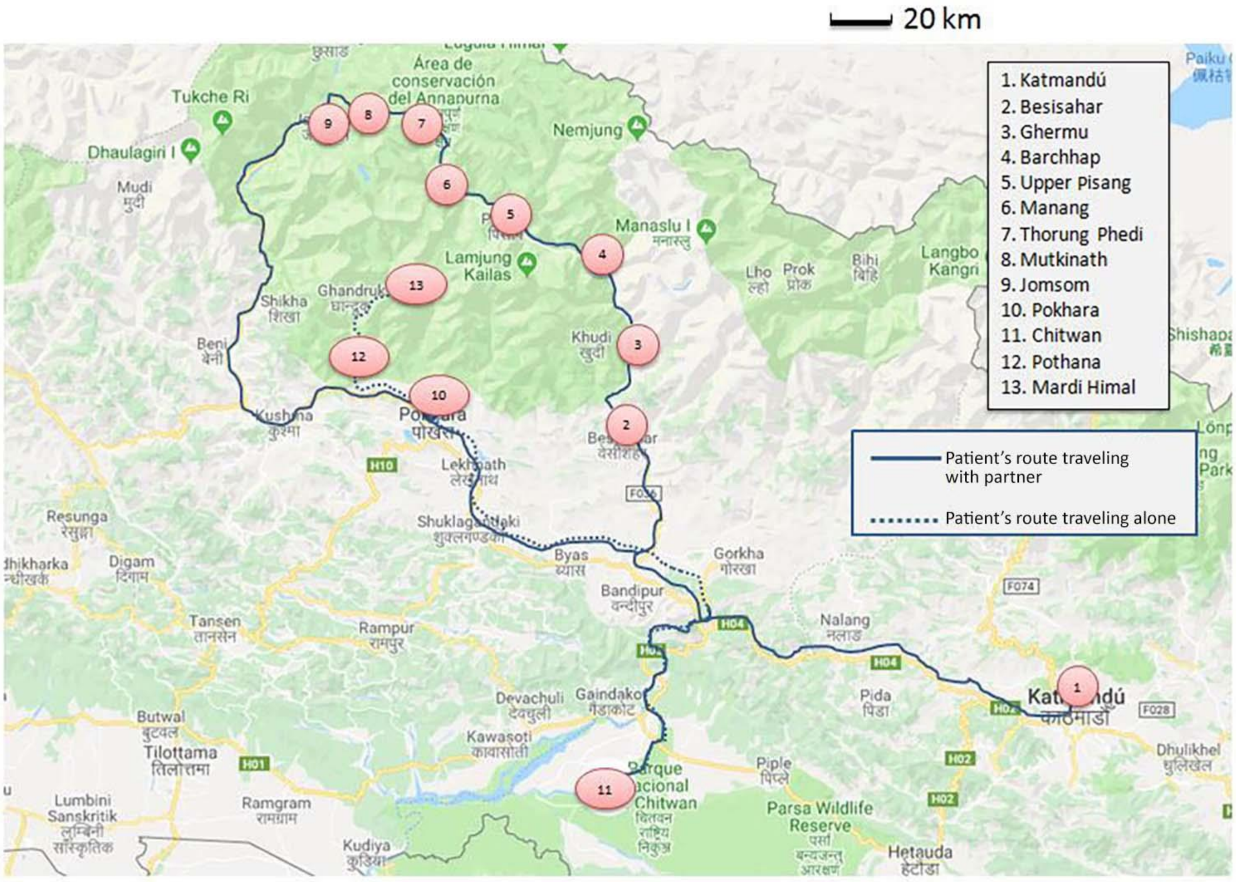

Appendix Figure. Travel route of a patient who returned from Nepal and was diagnosed with hantavirus pulmonary syndrome upon his return to Spain.
